# Anomalous origin of the left coronary artery from pulmonary artery mimicking antero-lateral ST-elevation myocardial infarction: a case report

**DOI:** 10.1093/ehjcr/ytae672

**Published:** 2024-12-18

**Authors:** Ziad Arow, Liaz Zilberman, Edward Koifman, Abid Assali, Yoav Arnson

**Affiliations:** Cardiology Department, Meir Medical Center, Tchernichovsky St 59, Kfar Saba 4418001, Israel; Sackler Faculty of Medicine, Tel-Aviv University, Kiryat HaUniversita, Ramat Aviv, Tel Aviv 6139001, Israel; Cardiology Department, Meir Medical Center, Tchernichovsky St 59, Kfar Saba 4418001, Israel; Sackler Faculty of Medicine, Tel-Aviv University, Kiryat HaUniversita, Ramat Aviv, Tel Aviv 6139001, Israel; Cardiology Department, Meir Medical Center, Tchernichovsky St 59, Kfar Saba 4418001, Israel; Sackler Faculty of Medicine, Tel-Aviv University, Kiryat HaUniversita, Ramat Aviv, Tel Aviv 6139001, Israel; Cardiology Department, Meir Medical Center, Tchernichovsky St 59, Kfar Saba 4418001, Israel; Sackler Faculty of Medicine, Tel-Aviv University, Kiryat HaUniversita, Ramat Aviv, Tel Aviv 6139001, Israel; Cardiology Department, Meir Medical Center, Tchernichovsky St 59, Kfar Saba 4418001, Israel; Sackler Faculty of Medicine, Tel-Aviv University, Kiryat HaUniversita, Ramat Aviv, Tel Aviv 6139001, Israel

**Keywords:** Case report, Anomalous coronary artery, Cardiac computed tomography, Echocardiography, Cardiac magnetic resonance

## Abstract

**Background:**

Anomalous origin of the left coronary artery (LCA) from the pulmonary artery (PA) (ALCAPA) is a rare congenital abnormality. We present a case of an ALCAPA in a 25-year-old man.

**Case summary:**

A 25-year-old male with no past medical history was admitted to our intensive cardiac care unit after sudden cardiac arrest due to ventricular fibrillation and suspected acute coronary syndrome. After being treated with shock by automated external defibrillator, an electrocardiogram (ECG) demonstrated sinus tachycardia with antero-lateral ST-segment elevation. Initial transthoracic echocardiography showed severe and diffuse left ventricular dysfunction and dilatation. Coronary angiography revealed anomalous origin of the LCA from the PA and extensive collateral circulation from a giant RCA. An ECG-gated cardiac computed tomography confirmed the diagnosis of anomalous left main originating from the left PA. Cardiac magnetic resonance demonstrated an enlarged left ventricle with globally reduced function and extensive sub-endocardial scarring of the anterior, antero-lateral, and lateral walls. Following a multidisciplinary heart team discussion, the patient successfully underwent repair of aberrant LCA with direct LCA re-implantation to the aorta and subcutaneous implantable cardioverter defibrillator implantation. Optimal medical therapy for heart failure with reduced ejection fraction was initiated, and the patient was discharged home for a close clinical and echocardiographic follow-up.

**Discussion:**

In conclusion, ALCAPA in the adulthood is a very rare congenital anomaly that clinicians should be aware of. The preferred treatment, when diagnosed in time, is direct re-implantation of the LCA to the aorta.

**Learning points**

- Anomalous origin of the left coronary artery from the pulmonary artery (ALCAPA) is a rare congenital abnormality associated with early infant mortality. Adults with ALCAPA may present with cardiac ischaemic symptoms or sudden cardiac death.
- Although invasive coronary angiography is the standard for ALCAPA diagnosis, non-invasive diagnostic testing (echocardiography, computed tomography, and MRI) plays a complementary role in identifying the coronary origins, left ventricular dilatation and dysfunction, and the extent and severity of the ischaemic cardiomyopathy. All these findings are essential for risk stratification, treatment, and follow-up.

## Introduction

Anomalous origin of the left coronary artery (LCA) from the pulmonary artery (PA) (ALCAPA) is a rare congenital abnormality that is associated with early infant mortality during the first year of life.^1^ Incidence is estimated at 1 in 300 000 live births. Only 10% of undiagnosed patients, with extensive collateral circulation from the right coronary artery (RCA), survive into adulthood. The average reported age (at diagnosis) in the adulthood is 41 years, with 2:1 predominance of females.^1–3^ Advanced cardiac imaging may facilitate the diagnosis of ALCAPA in the adult.^4,5^ We present a case of an ALCAPA in a 25-year-old adult.

## Summary figure

**Figure:**
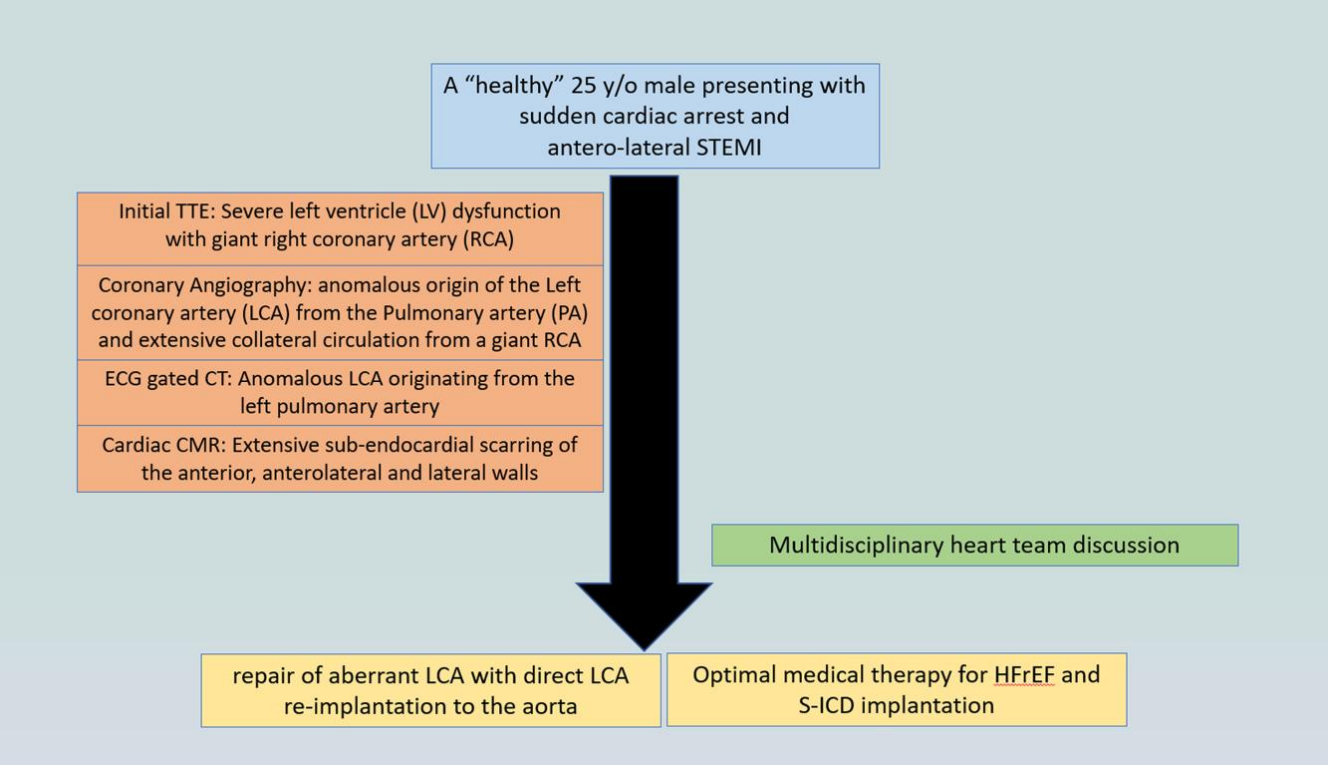


## Case presentation

**Figure 1:**
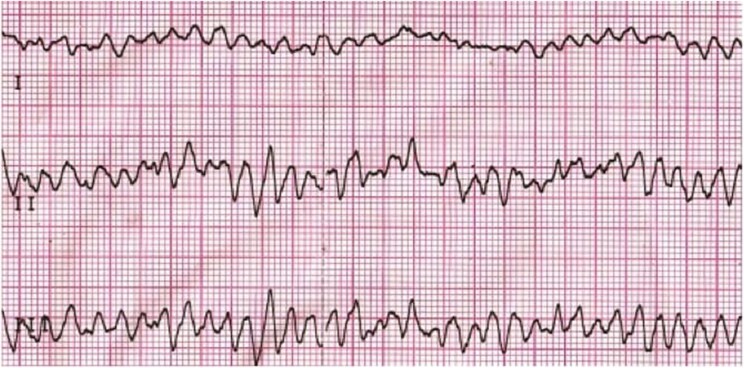
Electrocardiogram from automated external defibrillator.

**Figure 2:**
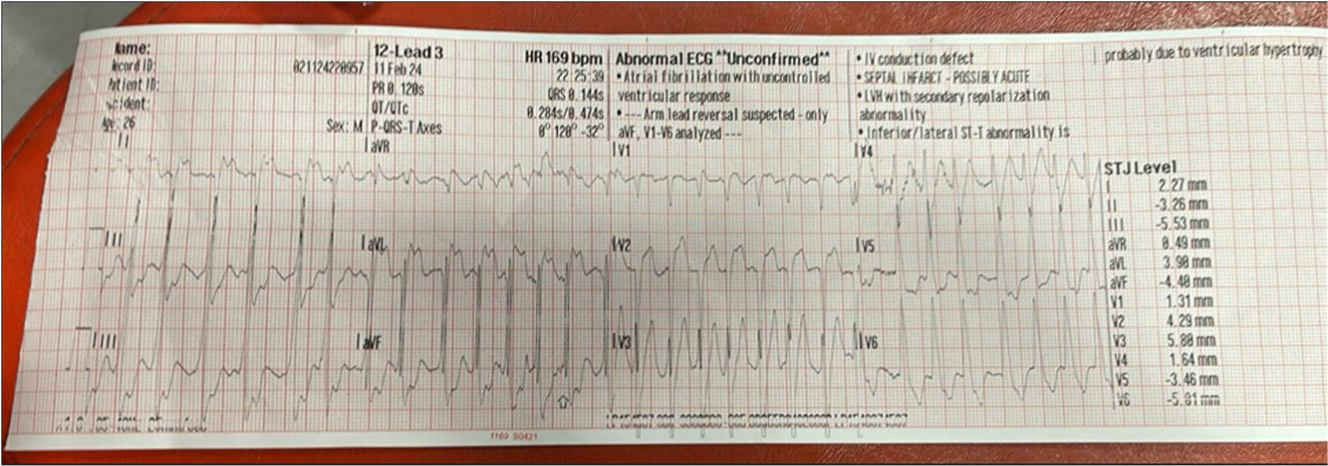
Electrocardiogram at initial presentation.

**Figure 3:**
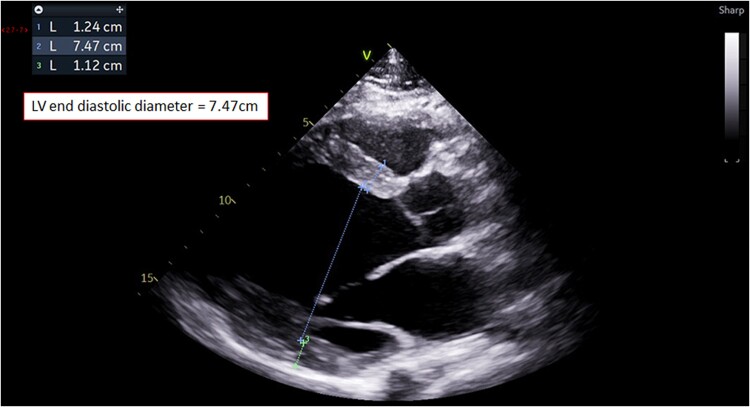
Transthoracic echocardiography—left ventricle diastolic dimensions.

**Figure 4:**
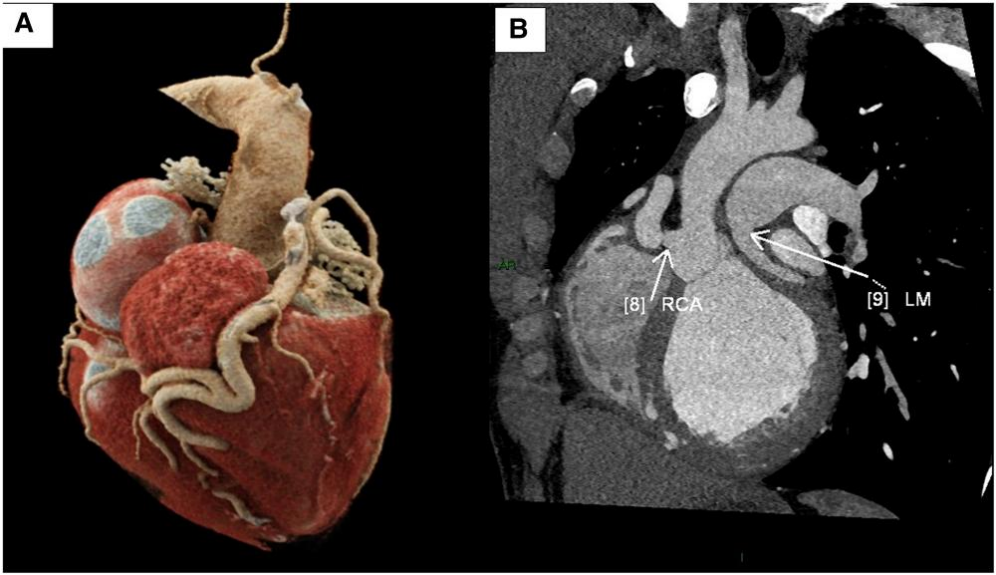
Computed tomography imaging of the coronary tree. (*A*) 3D rendering of the heart, showing the ectatic right coronary artery which supplies blood to the whole coronary tree. (*B*) A maximal intensity projection of the origins of the coronary arteries: the right coronary artery originating from the aorta and the left main originating from the left pulmonary artery.

**Figure 5:**
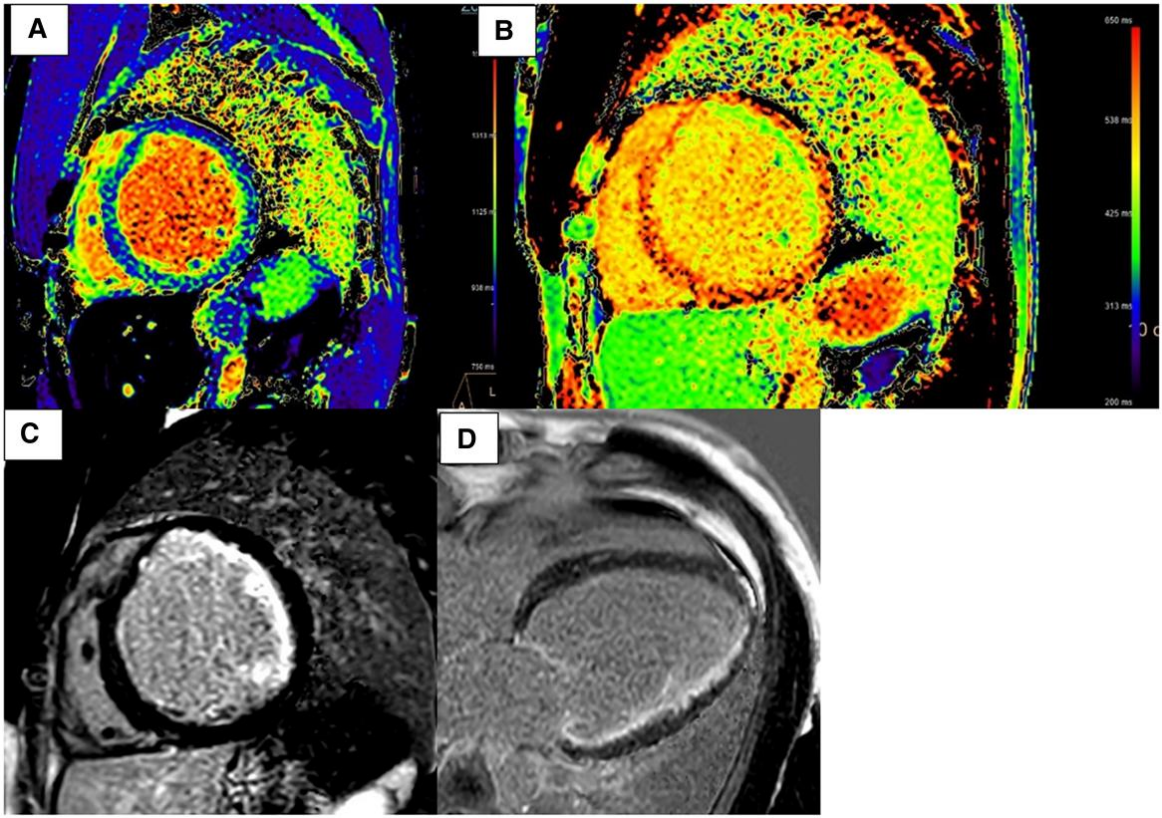
MRI imaging. MRI imaging showing extensive sub-endocardial scarring involving the papillary muscles, anterior and lateral segments. (*A*) Native T_1_ mapping with elevated T_1_ values. (*B*) Post-contrast T_1_ mapping, which shows sub-endocardial gadolinium uptake in the anterior and lateral segments. (*C*) and (*D*) show extensive late gadolinium uptake representing extensive sub-endocardial scarring.

A 25-year-old Arab male with no past medical history was admitted to our intensive cardiac care unit (ICCU) after sudden cardiac arrest due to ventricular fibrillation (VF) and suspected acute coronary syndrome. The patient never complained of symptoms suspected to be of cardiac origin and did not go any previous screening with electrocardiogram (ECG) or echocardiography. On the day of presentation, while working out at the gym (at higher level than usual), the patient suffered from sudden onset chest pain, palpitations, shortness of breath, and collapsed. The patient was immediately connected to automated external defibrillator pads and shock was delivered due to VF (*Figure 1*). Few minutes later, ECG was obtained by the paramedics arriving at the scene showing sinus tachycardia with antero-lateral ST-segment elevation and QS waves in leads V1, V2, I, and AVL (*Figure 2*). The patient was transferred urgently to the catheterization lab. Upon his arrival, the patient was haemodynamically stable but reported tightness in his chest; cardiac auscultation demonstrated regular and rapid heart sounds with no murmurs. Pulmonary crackles and elevated jugular venous pressure were noted with no peripheral oedema. Blood tests revealed normal levels of potassium, sodium, and magnesium. Initial transthoracic echocardiography (TTE) showed severe and diffuse left ventricular (LV) dysfunction and dilatation with giant RCA (Supplementary material online, *Videos S1* and *S2*; *Figure 3*). Coronary angiography revealed anomalous origin of the LCA from the PA and extensive collateral circulation from a giant RCA (Supplementary material online, *Video S3*), no coronary atherosclerosis or obstruction was noted, and LV gram showed severely dilated LV with severe and diffuse LV dysfunction (Supplementary material online, *Video S4*). The patient was transferred to the ICCU for further medical treatment and investigation. An ECG-gated cardiac computed tomography confirmed the diagnosis of anomalous left main originating from the left PA (*Figure 4*). Cardiac magnetic resonance demonstrated an enlarged left ventricle with globally reduced function, mild mitral regurgitation, and extensive sub-endocardial scarring of the papillary muscles, anterior, antero-lateral, and lateral walls. No right ventricle involvement, no other valvular dysfunction, or PA dilation was noted (*Figure 5*). During his hospitalization, the patient was constantly monitored with no evidence of recurrent cardiac arrhythmias. Following a multidisciplinary heart team discussion, including an invasive cardiologist, a heart failure specialist, an electrophysiologist, a cardiothoracic surgeon, and a cardiac imaging specialist, the patient successfully underwent repair of aberrant LCA with direct LCA re-implantation to the aorta. Post-surgical TTE still demonstrated severe LV dysfunction with estimated ejection fraction of 25%, and the patient successfully underwent a subcutaneous implantable cardioverter defibrillator (ICD) implantation (QRS width in post-surgical ECG was 110 ms with no indication for cardiac resynchronization therapy device). Finally, the patient was discharged home on maximally tolerated optimal medical therapy (OMT) for heart failure with reduced ejection fraction (HFrEF), including a beta-blocker, angiotensin receptor/neprilysin inhibitor, sodium-glucose cotransporter-2 inhibitor, and a mineralocorticoid receptor antagonist. Discharge home followed several detailed explanations to the patient and his family regarding his condition and a recommendation for a close clinical and echocardiographic follow-up. Patient reported no symptoms of heart failure during follow-up at the heart failure clinic, and echocardiographic follow-up demonstrated moderate LV dysfunction with estimated ejection fraction of 35%.

## Discussion

Anomalous origin of the left coronary artery from the pulmonary artery, also known as Bland–White–Garland syndrome, was reported for the first time in 1956 and represents the most common anomalous pulmonary origin of any coronary artery.^1,6^ Anomalous origin of the left coronary artery from the pulmonary artery is well tolerated *in utero* due to high pulmonary arterial pressure and saturation leading to antegrade flow in both the anomalous LCA and the normal RCA.^4,7,8^ Soon after birth and closure of the ductus arteriosus, the pulmonary arterial pressure and saturations gradually decrease, flow through the LCA reverses, and the oxygenated blood is shunted from high-resistance myocardial circulation to low-pressure pulmonary circulation (steal phenomenon) leading to poor perfusion with hypoxic blood of the anomalous LCA.^4^ The magnitude of collateral circulation developing from the RCA will determine the outcome^9,10^—death during the first months of life (in the majority of cases) or survival into adulthood (minority of cases). We present a rare case of ALCAPA presenting in the adulthood with VF, antero-lateral ST-segment elevation, and severe LV dysfunction. Classical ECG changes that should raise the suspicion for ALCAPA include Q-wave in lead aVL, ST-segment elevations in V3–V6, and negative T-waves in lead aVL, lead I, and precordial leads.^11–13^ The use of multimodality imaging and multidisciplinary heart team discussion was essential for accurate diagnosis and for further risk stratification and management of the patient. Interestingly, our patient remained asymptomatic for 25 years, most probably duo to compensated heart failure and by avoiding any daily physical work and practicing at low levels at the gym.

There are no specific guidelines as to how this complication should be managed, and the preferred treatment for ALCAPA, when diagnosed in time, is coronary artery bypass graft surgery or direct re-implantation of the LCA to the aorta.^14^ Patients with ALCAPA in the adulthood presenting with irreversible severe LV dysfunction should be offered OMT for HFrEF and ICD to prevent life threatening arrhythmias and sudden cardiac death.^15^

In conclusion, ALCAPA in the adulthood is a very rare congenital anomaly that clinicians should be aware of. The preferred treatment, when diagnosed in time, is direct re-implantation of the LCA to the aorta.

## Supplementary Material

ytae672_Supplementary_Data

## Data Availability

The data underlying this article will be shared on reasonable request to the corresponding author.

## Lead author biography

Ziad Arow is currently a resident in the Cardiology Department at Meir Medical Center in Israel. Ziad Arow finished his medical school (Technion, Israel) in 2016 and completed his internal medicine residency (Rabin Medical Center, Israel) in 2021.

## Notes

**Consent:** The authors confirm that written consent for the submission and publication of this case report, including images, videos, and associated text, was obtained from the patient in accordance with COPE guidance.

**Funding:** None declared.
